# *C-element*: A New Clustering Algorithm to Find High Quality Functional Modules in PPI Networks

**DOI:** 10.1371/journal.pone.0072366

**Published:** 2013-09-05

**Authors:** Mahdieh Ghasemi, Maseud Rahgozar, Gholamreza Bidkhori, Ali Masoudi-Nejad

**Affiliations:** 1 Laboratory of Systems Biology and Bioinformatics (LBB), Institute of Biochemistry and Biophysics, University of Tehran, Tehran, Iran; 2 Database Research Group (DBRG), Control and Intelligent Processing Center of Excellence (CIPCE), School of Electrical and Computer Engineering, College of Engineering, University of Tehran, Tehran, Iran; University of Rome, Italy

## Abstract

Graph clustering algorithms are widely used in the analysis of biological networks. Extracting functional modules in protein-protein interaction (PPI) networks is one such use. Most clustering algorithms whose focuses are on finding functional modules try either to find a clique like sub networks or to grow clusters starting from vertices with high degrees as seeds. These algorithms do not make any difference between a biological network and any other networks. In the current research, we present a new procedure to find functional modules in PPI networks. Our main idea is to model a biological concept and to use this concept for finding good functional modules in PPI networks. In order to evaluate the quality of the obtained clusters, we compared the results of our algorithm with those of some other widely used clustering algorithms on three high throughput PPI networks from *Sacchromyces Cerevisiae, Homo sapiens and Caenorhabditis elegans* as well as on some tissue specific networks. Gene Ontology (GO) analyses were used to compare the results of different algorithms. Each algorithm's result was then compared with GO-term derived functional modules. We also analyzed the effect of using tissue specific networks on the quality of the obtained clusters. The experimental results indicate that the new algorithm outperforms most of the others, and this improvement is more significant when tissue specific networks are used.

## Introduction

Graph clustering of PPI networks is one of the most common techniques for inferring functional modules [1]–[5]. Although there is no widely accepted formal definition of a functional module, it is commonly conceived as a group of proteins that work together to carry out a cellular process while binding to each other in different times and places [6].Various graph clustering approaches have been developed in order to discover sets of densely connected vertices within a graph. Graph clustering approaches can be categorized into several categories such as *graph partitioning*, *hierarchical clustering*, *partitional clustering*, *spectral clustering* and *modularity optimization based clustering* [7]. Most clustering algorithms with the purpose of finding functional modules in bioinformatics, try to find either highly dense sub graphs based on finding cliques or in a greedy manner grow clusters starting from vertices with high degrees as seeds. A clique is a complete graph in which all the vertices are directly connected to each other. Since PPI networks are typically sparse [8], clique based methods only can find small number of clusters that only cover scant number of proteins and omit lots of proteins in PPI network. For example in our analysis *CFinder* [9] clusters only 596 proteins out of 2305 in *Yeast* PPI network, 826 proteins out of 3726 in Human PPI network and 28 proteins out of 305 in *C.elegans* PPI network [10], [11]. In this paper, we present a new algorithm in order to find functional modules in PPI networks utilizing the advantages of these both categories. The algorithm consists of two main parts. The main goal of the first part is to determine the best seeds by finding and removing the special proteins that might belong to the several clusters and consequently to find the best clusters. The main idea of the second part is to take the profits of clique-based clustering algorithms. To demonstrate the ability of the new algorithm to find the functional modules, we set up our experiments using GO analyses on the high throughput PPI networks such as *Saccharomyces Cerevisiae, Homo sapiens and C.elegans* and also on tissue specific PPI networks. We use *GO* Biological Process (*BP)* and *GO* Cellular Component (*CC)* derived functional modules to evaluate the power of the new algorithm and some other clustering algorithms in finding functional modules in PPI networks. The GO analyses are based on the framework introduced by Song and Singh [12]. We also analyze the effect of using tissue specific networks on the quality of different algorithms' results.

### Background and related work

There are lots of graph clustering algorithms proposed up to now. Here, we briefly explain those methods whose focuses are on finding functional modules in PPI networks. Some of the algorithms such as *CFinder* [9] try to find clique like sub networks. These algorithms can only cluster a small number of vertices in the PPI networks because PPI networks are completely sparse [8]. In 2006, Adamcsek et al introduced *CFinder* [9] as a tool to find highly inter connected area in graphs based on Clique Percolation Method (*CPM*) [13]. *CFinder* finds all k-cliques that are defined as complete sub-graphs with k vertices (k≥3), and then it merges k-cliques if they share exactly k-1 vertices. The other algorithms such as *SPICi* [14] and *clusterONE* [15] optimistically suppose that the clusters in PPI networks are located around the vertices with high degrees. These algorithms make clusters starting from these vertices as seeds. Jiang et al.in 2010 presented *SPICi*, a runtime and memory efficient clustering algorithm. *The spice* is a greedy, runtime and memory efficient clustering algorithm that makes cluster, starting from vertices with high degrees (hubs) as seeds and grows them locally in order to optimize cluster's density. *ClusterONE* is a clustering algorithm that generates overlapping clusters by growing clusters with high cohesiveness from selected seed vertices. This algorithm is another seed-based clustering algorithm that was presented by Nepusz et al. in 2012. *MCODE* is a popular clustering algorithm to detect densely connected regions in large PPI networks [1]. *MCODE* was introduced by Bader et al. in 2003 and it entails three main steps, 1) vertex weighting: *MCODE* weights all vertices based on their local network density, 2) complex prediction: starts from a vertex with the highest weight as a seed and grows clusters by iteratively adding seed's neighbors whose weight is above a given threshold. When no more vertex can be added to current cluster, *MCODE* repeatedly starts from the next highest unseen weighted vertex in the network, and 3) post processing to filter or add proteins in the resulting complexes. Step Three is optional. Corban et al. in 2010 introduced *NeMo* as a new clustering algorithm [16]. *NeMo* computes a log-odds score [17], [18] of shared neighbors for all pairs of vertices and then uses hierarchical agglomerative clustering using either single-linkage or complete-linkage clustering to make final clusters. In 2011, Rhrissorrakrai et al. presented *MINE* [19] whose strategy is similar to that of *MCODE*. In [19] Authors argued that *MCODE* gives a good result on the *Yeast* PPI network, but it does not produce a good clustering result on *C. elegans* PPI network. The primary difference between *MINE* and *MCODE* is their weighting functions. *MINE* also uses *modularity* optimization strategy. For each vertex v, *MINE* assigns weight that is the product of v's clustering coefficient [20] and the number of edges (k) of the most highly connected node in the local neighborhood of *v*. This weighting function assigns higher weight to those vertices that are connected to a vertex with high degree (hubs). Enright et al introduced *MCL* in 2002 [21]. *MCL* is based on the idea that if a random walker starts from a vertex and randomly chooses an edge to continue its traveling; it is more probable that it stays within a cluster than travels to another one. This happens simply because there will be more edges within a cluster than edges between clusters and then most likely an inside edge will be chosen. The focus of these briefly described algorithms is on finding functional modules in PPI networks. There are also some other graph clustering algorithms. Fortunato et al. [22] argued that algorithms based on optimizing *modularity* suffer from a resolution limit. *Modularity* is a quantitative measure that was originally defined by Newman and Girvan [23], to assess the quality of graph clustering results. Based on this measure, a clustering algorithm gets high modularity if it finds clusters with dense connections between the each cluster's inside and gets low modularity scpre with sparse connections. Resolution limit means small scale clusters or clusters with high density may be merged into a single larger community. To solve this problem Ruan el al. in 2008, introduced two new algorithms *QCUT* and *HQCUT* [24]. *QCUT* is a heuristic algorithm based on optimizing modularity. *QCUT* consist of two steps. First it uses spectral graph partitioning to divide the input graph and next attempts to merge or change the result of previous step in order to optimize modularity. To solve the resolution limit problem Ruan el al. suggested recursively applying *QCUT* on subnetworks that are found with *QCUT* while ignoring all the inter clusters edges. They called this procedure *HQCUT*. *FAG_EC* [25] is a *Hierarchical agglomerative* algorithm that was introduced by Li et al. in 2008. *FAG_EC* identifies modules based on edge weighting function, (called) *edge clustering coefficients*. It sorts all edges based on their edge clustering coefficients descending. Repeatedly choose an edge with the highest weight and decides whether or not the two edge's endpoints are in the same cluster or not.

In the rest of this paper, we present a new algorithm called *C_element* and the extended version of this algorithm (*C_element_extended*) to find functional modules in PPI networks. The main idea of this algorithm is to improve the quality of obtained clusters utilizing the benefits of both clique-based and seed-based algorithms.

## Materials and Methods

In this section, we first briefly explain some basic concepts and primary definitions related to the proposed algorithm and then we explain the algorithm in more details.

### Preliminaries

A graph is a representation of a set of objects where some pair of these objects is connected via a link. Therefore, a graph *G (V, E)* consists of two sets V and E where V is the vertex set and E is the edge set [26]. PPI networks can be modeled as a graph. Given a PPI network, the goal of the proposed algorithm is to find internally high connected sub graphs (clusters) in order to extract functional modules in PPI networks. In this paper, networks are considered as an un-weighted and undirected graph.

### The proposed algorithm

**Figure 1:**
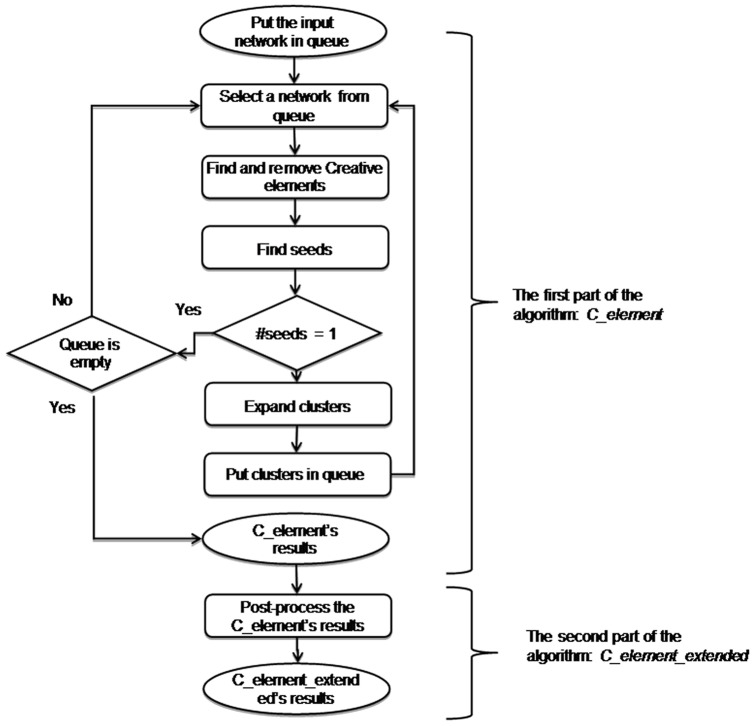
An overview of the proposed algorithm; the algorithm consists of two main parts.

The proposed algorithm consists of two main parts. The main goal of the first part is to find the best seeds by finding and removing the special proteins that might belong to the several clusters and consequently to find the best clusters. We expect that the result of this part outperforms the results of the seed-based algorithms such as *SPICi* and *clusterOne*. The main idea of the second part is to take also the profits of clique based clustering algorithms as a complementary solution. In the rest of this section, we explain each part in more detail. Figure 1 shows an overview of the proposed algorithm.

### The First part of the algorithm: *C_element*

**Figure 2:**
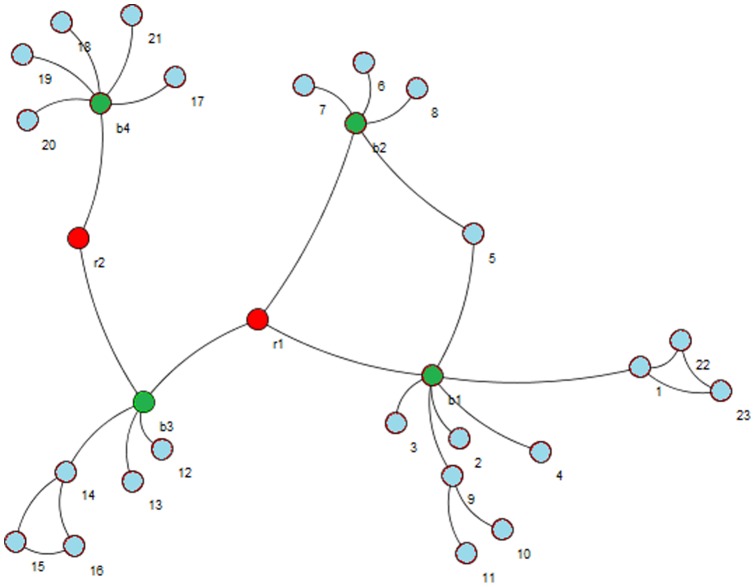
Two red vertices A and A' are creative elements and green vertices are hubs. The original network can be found in [27].

In biology a protein segment is called an *active site* if it plays a key role in the catalytic action of the enzyme function and substrates bind and undergo a chemical reaction. Csermely proposed that the concept of active sites can be extended to networks other than protein structures [27]. He mentioned that the active sites of networks (for example PPI networks ) at higher levels than proteins are not only central elements with a specific set of properties and with ability to monitor all the communications of the entire network, but they are also randomly scattered over the whole network while connecting distant modules. Active sites have weak connections to important vertices (often known as hubs) in the network. Csermely proposed the term *creative elements* for the vertices that are located in active sites. A sample network with two *creative elements* is shown in Figure 2. This network is clipped from Csermely's original paper [27].

Our main idea is that by discovering and removing *creative elements* (vertices) from the network, the modules in that network would appear clearly.

**Figure 3:**
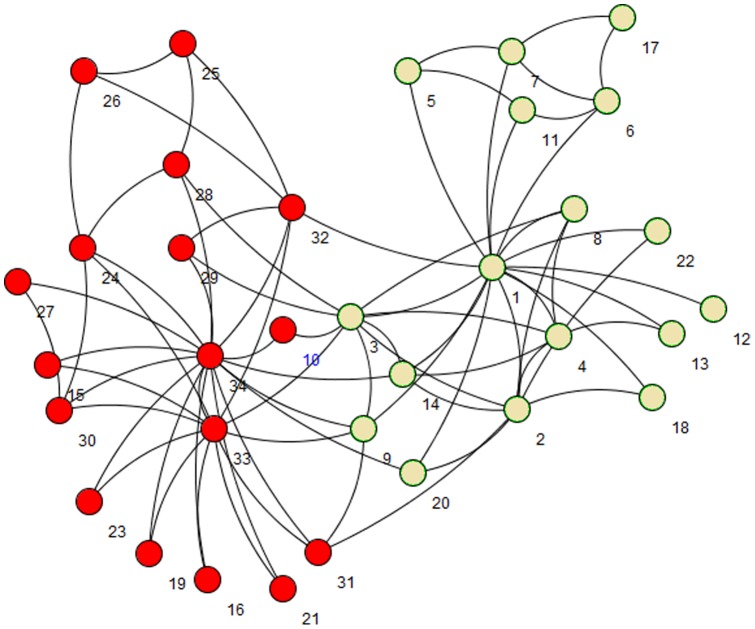
Zachary's karate club network; two main clusters indicates by different colors.

Using *Centrality measures* is an idea to find *Creative elements*. *Centrality measures* are functions that assign numerical value to each vertex in the network in order to find more important vertices. Based on these values, the vertices in the network can be ranked. Based on these measures, the more influential vertices are those get higher scores. In order to assay the power of *Centrality measures* to find *Creative elements* we simply applied some of these measures on the sample network represented in Figure 2 and also on Zachary's well-known “karate club” network [28]. The karate club network is a standard benchmark in community detection algorithms [7]. The karate club network is visualized in Figure 3. Our main reason to choose this social network rather than any other biological networks is its popularity and being well studied in researches on graph clustering. Unfortunately, there is not any standard biological network benchmark on graph clustering. The karate club network consists of 34 vertices and 78 edges. The vertices in that network are labeled from 1 to 34. Two main clusters in karate club are shown in different colors in Figure 3. Table 1 briefly shows the *Centrality measures* that we used to analyze the both mentioned networks.

**Table 1:** The Centrality Measures that used to find Creative elements.

Measure	Equation/Definition	Reference
Degree centrality	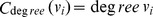	Koschützk et al. [29]
Leverage centrality	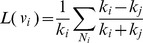	Joyce et al. [30]
Local leader	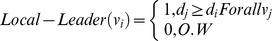	Blondel et al. [31]
Strict leader	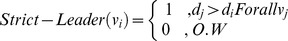	Blondel et al. [31]
Closeness	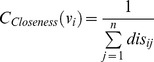	Sabidussi[32]
Eccentricity	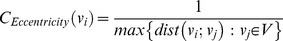	Hage et al. [33]
Radiality	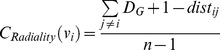	Valente el al. [34]
Shortest-path betweenness	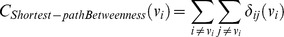	Freeman [35]
PageRank	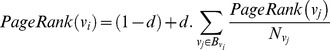	Page et al. [36]
Eigenvector	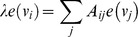	Bonacich[37]
Power	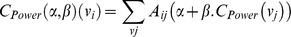	Bonacich[37]
Cluatering coefficient	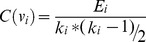	Watts et al. [20]
K-step markov	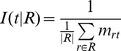	White et al. [38]

We applied all the measures in Table 1 on the both networks. Table 2 briefly shows the obtained results. We simply show some top-ranked vertices based on each measure.

**Table 2:** The top-ranked vertices in two networks based on different centrality measures.

Measure	The top-ranked vertices in the network from Figure 2 (A)	The top-ranked vertices in karate club
**Degree centrality**	b1, b3, b4	34, 1, 33, 3, 2, 32, 4, 14, 24, 9
**Leverage centrality**	b2, b3, 9	7, 30, 33, 32, 3, 1, 28, 6
**Local leader**	b1, b2, b3, b4	34, 1
**Strict leader**	b1, b2, b3, b4	34, 1
**Closeness**	b1, b3, r1	1, 3, 34, 32, 33, 14, 9, 20
**Eccentricity**	b1, b3, r1	1, 2, 3, 4, 9, 14, 20, 39
**Radiality**	b1, b3, r1	1, 3, 34, 32, 9, 14, 33, 20
**Shortest-path betweenness**	b1, b3, r1	1, 34, 33, 3, 32, 9, 2, 14
**PageRank**	b1, b3, b4	34, 1, 3, 33, 2, 9, 14, 4
**Eigenvector**	b1, r1, 5	34, 1, 3, 33, 2, 9, 14, 4
**Power**	b1, b4, b2	34, 1, 25, 26, 17, 33, 2, 12
**Clustering coefficient**	15, 16, 22, 23	19, 17, 18, 15, 16, 13, 21, 22, 23, 27, 8
**K-step markov**	b1, r1, 14	1, 34, 33, 3, 2, 32, 4, 14, 9

After analyzing Table 2, we realized that all those retrieved vertices have somehow important role in corresponding networks. For example, the main hubs of both networks are given the highest scores by measures *Local leader*, *Strict leader* and *Degree centrality* as well. The results of each measure on mentioned networks are available as supplementary data attached to this paper (File S1 and File S2) or following link: http://lbb.ut.ac.ir/Download/LBBsoft/C-elemnts-Clustering/.

Although, these measures marked some vertices in the network as important vertices that might have a key role in the network, none of them could fulfill our goal to find the *creative elements* vertices (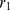 and 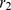). In order to find these *creative elements* we propose equation (1).
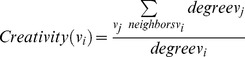
(1)Where 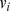 belongs to a set of vertices whose neighbor set contains at least one hub. Here hubs refer to vertices with high degree. We consider 20% top vertices based on their degrees as hub. If a vertex 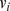 has several hubs in its neighborhood and its degree is low, it will get a high 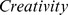 score. The higher score each vertex gets, the more creative it is. Equation (1) can be considered as a new *Centrality measure*.

**Figure 4:**
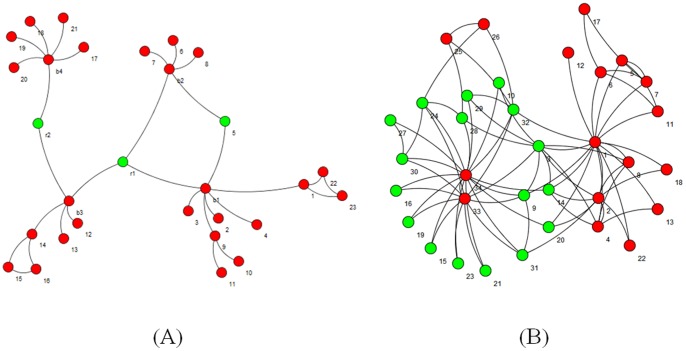
Green vertices are the most creative elements realized by equation (1) in a network which is illustrated Figure 2 (A) and in Zachary's karate club network (B).

**Figure 5:**
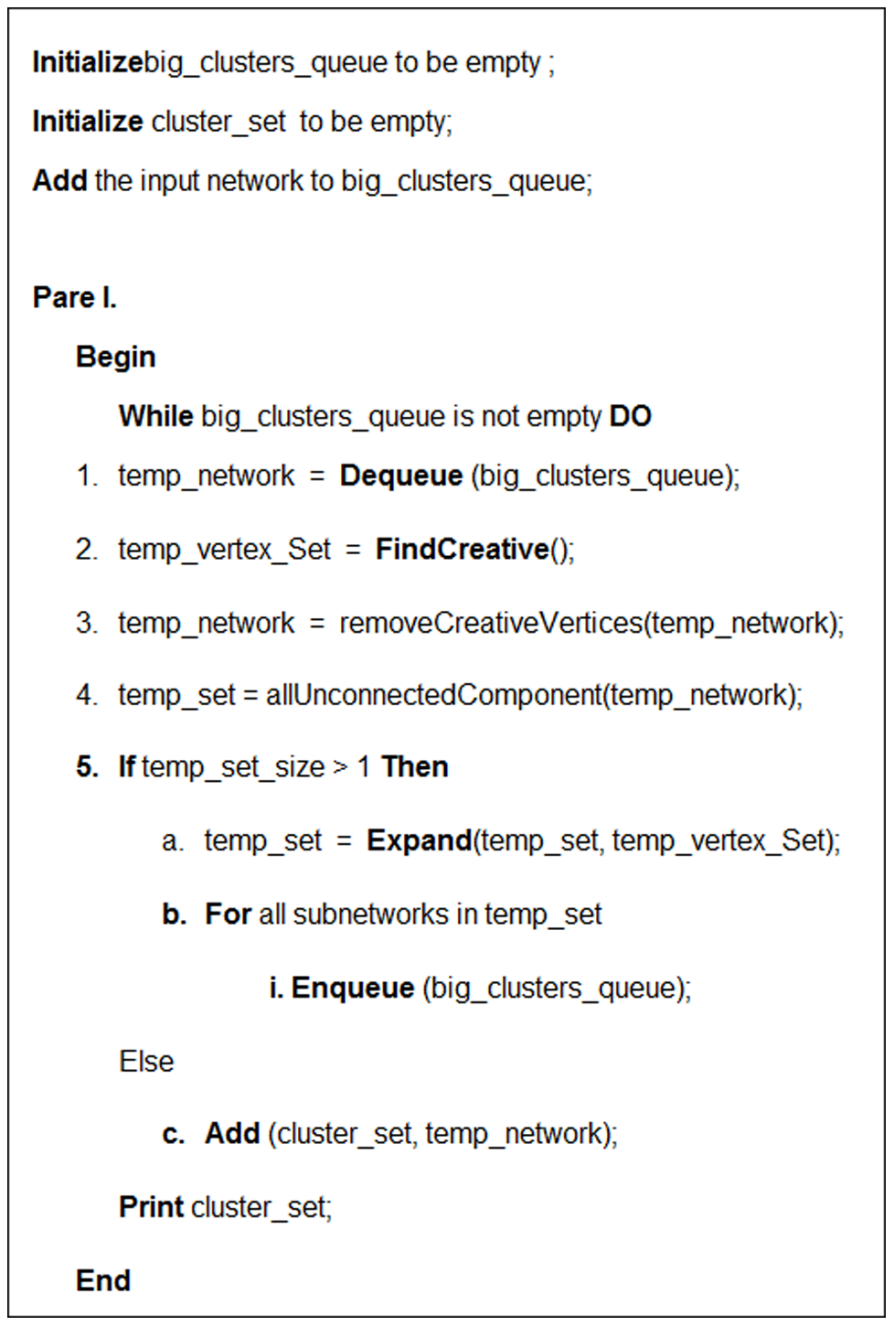
The pseudocode of the first part of the proposed algorithm.

We applied equation (1) on the network in Figure 2 in order to check its ability to find *Creative elements*. Figure 4 (A) is visualized the results. The three vertices with the highest *Centrality* scores are colored in green. As it can be realized clearly, two *creative elements* mentioned in Figure 2 are among these three green vertices. We also applied equation (1) on karate club. Green vertices in Figure 4 (B) are more creative vertices based on equation (1). We realized that many of these vertices such as 3, 14, 20, 31 and 9 are located on the border between two main clusters; that means these vertices have connections to other vertices in two main clusters. This fact is used in the first part of the new algorithm in order to find a good seed set and consequently to find good clusters in PPI networks. The pseudo code of the first part of the algorithm is presented in Figure 5.

- At the beginning of the algorithm, there is a queue of big clusters (*big_clusters_queue*) waiting to analyze and also there is an empty set of founded clusters (*cluster_set*). Initially, all the vertices in the network in hand make the cluster at the top level of hierarchy. This means algorithm starts with *big_clusters_queue* with one member, the whole input network.
- One of the sub-networks in *big_clusters_queue* is chosen and is removed from the queue. *Creative elements* in this sub-network will be found and will be removed in order to separate existed clusters in that sub-network. These vertices are supposed to be located on the borders between clusters. After removing the *Creative elements* and all the Interactions between these elements and any other vertices in the sub-network, the remaining vertices and interactions in the sub-network might make an unconnected network. In this step all the unconnected components of the leftover sub-network is obtained. These components are a collection of some singleton vertices or groups of connected vertices that are made *C_elements* beginning seeds. Then the algorithm expands these seeds to make the clusters using the Expand function. There is not an assumption about the number of clusters that must be found in each step. ­After removing the *Creative elements*, if the leftover sub-network remains connected, it is not clusterable and it is one of the clusters at the lower level of the hierarchy. This means this sub-network do not have any sub-clusters. In this situation *C_element* adds this sub-network to *cluster_set* and continues processing another subnetwork in the *big_clusters_queue.*
- The Expand function assigns the removed *creative elements* to one of the obtained seeds in a greedy manner. This seed is one that maximizes the equation (2).
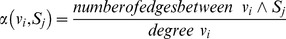
(2)Where 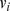 is one of the creative elements and 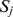 is a founded seed of the previous step. For each removed creative element 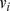, the Expand function search for the seed 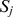 with maximum value of 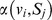 among all the obtained seeds that are found in the previous step. Based on this equation, a vertex assign to a growing cluster in which its most neighbors located. All the resulting clusters are added to *big_clusters_queue* for further analyses.
- This process continues until there is not any other subnetwork in the *big_clusters_queue*. In that case the final clusters are in *cluster_set.*

### The second part of the algorithm

**Figure 6:**
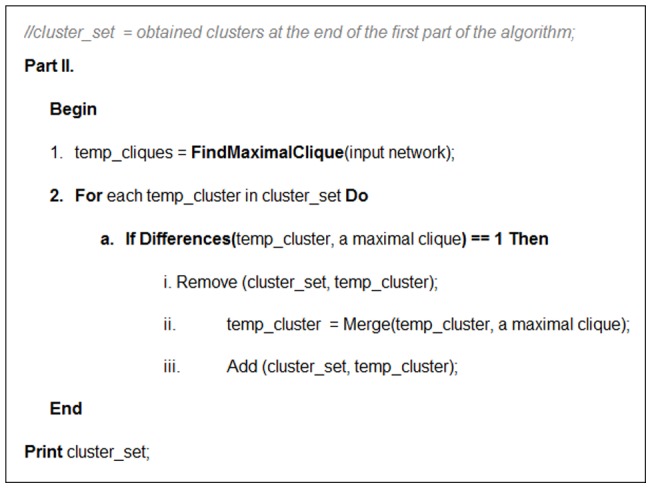
The pseudo code of the second part of the proposed algorithm.

In the second part of the algorithm the advantages of clique based clustering algorithms are utilized. The pseudocode of the second part of the algorithm is presented in Figure 6. In the second part, the algorithm finds all maximal cliques with size≥3 in the original input PPI network. A clique is called a maximal clique if it is not part of a bigger clique [6].The algorithm merges each *C_element*'s already obtained cluster with n vertices and a maximal clique into one new cluster if they have n-1vertices in common. If a cluster (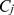) and a maximal clique are merged, they make a new larger cluster. Then 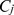 is replaced by that larger cluster. By using this post-processing step, some small *C_element*'s output clusters that have low quality are merged with a maximal clique and the overall quality of the obtained clusters is outperformed. Using this post-processing step, the number of *C_element*'*s* final clusters might decrease. We refer to the resulting final clusters at the end of this part as *C_element_extended.*

Our new algorithm, *C_element*, belongs to *Hierarchical Divisive algorithm* category. *Hierarchical clustering* approach can be classified in two categories: 1) *Agglomerative algorithms:* this is a *bottom-up* approach; at the first step starts with singleton clusters which each vertex makes a cluster with only one vertex belong to and iteratively clusters at the higher level of hierarchy are merged if their similarity is high enough; 2) *Divisive algorithms:* this is a *top-down* approach; at the first step all vertices belong to one big cluster, and recursively clusters at the higher level of hierarchy are split and make the next level. One of the advantages of *Hierarchical clustering* approach is that it does not require a preliminary knowledge of the number and size of the clusters [7].

### Datasets

We applied the new algorithm to high throughput PPI networks from *Sacchromyces cerevisiae* (*Yeast*), *Homo sapiens* (*Human*) and *C.elegans* (*Worm*) that are obtained by Patil and Nakamura [10], [11]. They categorized the interactions into three categories: High confidence small-scale binary interactions, High confidence interactions based on reliability score and Low confidence interactions based on reliability score. All these interactions are available online on the project's website [39]. In this paper, we use high confidence small-scale binary interactions for each mentioned species as their corresponding PPI networks.

*Sacchromyces cerevisiae* PPI network consists of 2324 proteins connected via 4376 interactions, Homo *sapiens* PPI network consists of 3989 proteins connected via 7465 interactions and *C.elegans* PPI network consists of 379 proteins connected via 385 interactions.

We also applied our algorithm to tissue specific human PPI networks [40]. These tissue specific networks consist of 60 PPI networks for 60 specific tissues in the human body. In this paper we show the results of applying different algorithms on *B cells, Bone marrow CD34* and *Monocytes* PPI networks. The *B cells* PPI *network* consists of 942 proteins connected via 2026 interactions, *Bone marrow CD34* PPI network consists of 1669 proteins connected via 4552 interactions and *Monocytes* PPI network consists of 1549 proteins connected via 4025 interactions.

### GO analysis

In order to evaluate the power of our clustering algorithm in finding functional modules in PPI networks, we utilize the framework which is described by Song and Singh [12]. This framework quantifies how well computationally derived clusters in physical interactomes correspond to functional modules derived via the *Gene Ontology* (*GO*) [41]. Each *GO* term generates a functional module that means all the proteins that are annotated with a same *GO* term correspond to same functional modules.

Song and Singh [12] utilized the following three formulas to quantify the overlap between computationally derived clusters and *GO* derived functional modules:

1) Jaccard measure:

For each computationally derived cluster 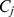 *jaccard similarity coefficient* is defined as the maximum value 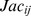 over all the functional modules 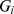 that are extracted by considering different *GO* terms *A*. 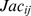 between cluster 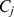 and module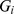 is defined as the size of the intersection over the size of the union. Equation (3) shows this measure.
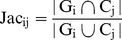
(3)


2) Precision–Recall measure:

For each cluster 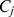, its *Precision–Recall* (*PR*) value with a *GO* derived functional module 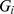 is computed using equation (4). The final *PR* measure for 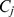, is the maximum *PR* value over all 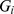
s.
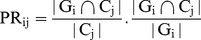
(4)


3) Semantic density measure:

This measure measures the average *semantic similarity* between each pair of annotated proteins within a cluster and can be calculated using equation (5).
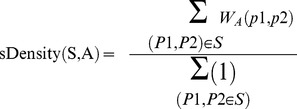
(5)Where S is a cluster, 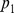 and 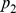 are two proteins belong to S, A is a set of all *GO* derived functional modules and 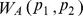 is a weight function that assigns a weight to a pair of proteins 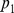 and 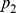 in the range of 0–1. 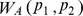 is calculated using equation (6).
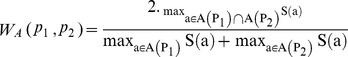
(6)Where 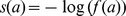 shows how specific the annotation 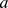 is and 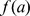 is the fraction of the total number of proteins in the considered network that have annotation 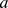.

Each of these three measures varies from 0 to 1. The higher values for each clustering result mean the better agreement of the clusters on *GO* derived functional modules. Further information on *GO* analyses can be found on [12].

## Results and Discussion

### Go analysis

We used the *GO* biological process (*BP*) and cellular component (*CC*) terms to extract the functional modules. Molecular function *GO* terms (*MF*) were not used because proteins annotated with the same *GO MF* terms do not necessarily interact with each other as a functional unit [12].We considered the *GO BP* and *GO CC* ontologies separately. For each cluster, we separately calculated the three measures introduced in the previous section for both ontologies. For Genes in singleton clusters, we assigned *Jaccard, PR* and *semantic similarity* values of 0. Finally, each cluster was weighted by its size, and the final values for each of the six measures (three *BP* and three *CC*), was computed using the weighted average over all clusters in the computational clustering result. We compared our algorithm with 5 other popular clustering algorithms. Some clustering algorithms like *MCODE* [1] finds small number of very good clusters and omits from consideration lots of vertices. For example in our analyses, in *Yeast* PPI network, all number of vertices was 2305 and *MCODE* only clustered 244 proteins out of 2324 proteins and left 2061 proteins without assigning them to any clusters. Also in our experiments quality of some clustering algorithms such as *FAG-EC* [25], *QCUT* and *HQCUT* [24] was related to the input network. For example in our analyses *QCUT* and *HQCUT* found good resulting clusters on *Homo sapiens*, but they could not find any clusters on *C.elegans* and *Yeast* PPI networks. Therefore, in our analyses we focused on *clusterONE* (2012) [15], *SPICi* (2010) [14], *NEMO* (2010) [16]; *CFinder* (2006) [9] and *MINE* (2011) [19] that showed stable behavior on different networks. Implementations of each of these algorithms are available either on Cytoscape website [42] or on each related project's website.

### Jaccard measure analyses

**Figure 7:**
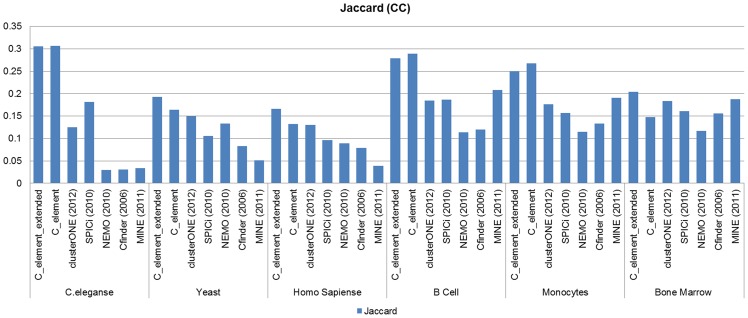
The results of Jaccard measure analyses on the results of different algorithms on different network datasets when *GO CC* derived functional modules were considered.

**Figure 8:**
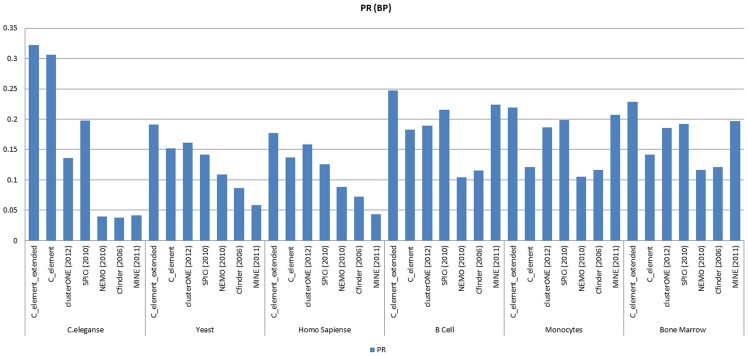
Jaccard measure analyses results on the different network datasets and different algorithms when *GO BP* derived functional modules were considered.

Figure 7 and Figure 8 show the results of *Jaccard* measure analyses on the results of different algorithms when *GO CC* derived functional modules and *GO BP* derived functional modules were considered as the reference set of known modules respectively.

As it can be inferred from the corresponding diagrams in Figure 7 when *GO CC* derived functional modules were considered and based on J*accard* measure analyses, the first part of the proposed algorithm (*C_element*) outperformed other algorithms in all three high throughput PPI networks and found that the *GO CC* derived functional modules were better than the other algorithms. Especially the result of the new algorithm on *C.elegans* PPI network was considerable. *C_elemnt* also acted well on tissue specific networks. Bone marrow was the only network on which *clusterOne* got higher *Jaccard* scores than *C_element*. Generally, the *clusterONE* clustering algorithm also found good clusters and surpassed the other four algorithms in most cases. The only case in which the *SPICi* clustering algorithm got better results than *ClusterONE* was on *C.elegans* PPI network, and in all the other cases our algorithm and *ClusterONE* seemed to have foundbetter *GO CC* derived functional modules than the other algorithms. After merging the clustering results of *C_element* with maximal cliques, the quality of the clusters was surprisingly improved and *C_element_extended* all the other algorithms.

### Precision–Recall analyses

**Figure 9:**
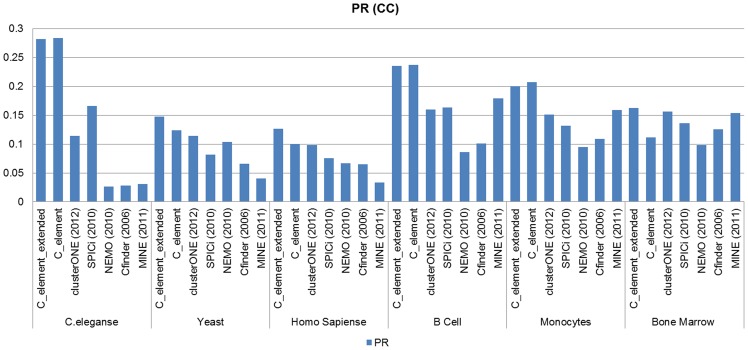
The results of Precision–Recall measure analyses on the results of different algorithms on different network datasets when *GO CC* derived functional modules were considered.

**Figure 10:**
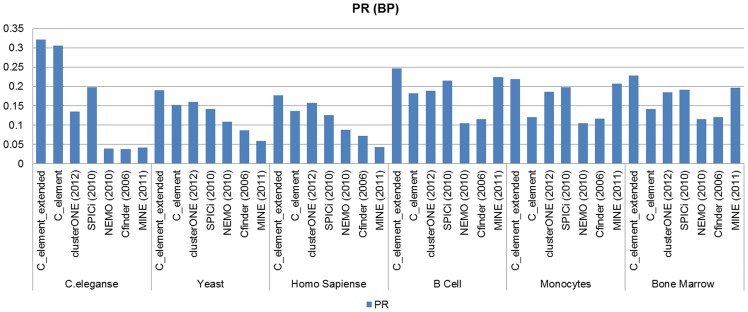
The Precision–Recall measure analyses results on the results of different algorithms on the six different network datasets when *GO CC* derived functional modules were considered.

Figure 9 and Figure 10 show Precision–Recall measure analyses results on the results of different algorithms when *GO CC* derived functional modules and *GO BP* derived functional modules were considered as the reference set of known modules respectively. Our Precision–Recall measure analyses verified the obtained results by *Jaccard* measure. As it can be realized clearly from the corresponding diagrams, the success of our algorithms (both *C_element* and *C_element_extended*) to find *GO* derived functional modules in *C.elegans* PPI network and also on *B cells* PPI network was undeniable in comparison to other algorithms. In *Yeast* and *Human* PPI networks by considering *GO BP* derived functional modules there was a close competition between the first part of the proposed algorithm and *clusterONE*; that means on some input networks such as *Bone Marrow CD34 clusterONE* outperformed *C_element*and on some input network datasets such as *C.eleganse*, *B Cells* and *Monocytes C_element* outperformed *clusterONE*. In all cases *C_element_extended* outperformed other algorithms. In summary, Precision–Recall measure analysis on *B cells, Bone marrow CD34* and *Monocytes* tissue specific PPI networks confirmed previous *Jaccard* analyses. *MINE* and *SPICi* showed good results in finding *BP* derived functional modules and outperformed *clusterONE* and *C-element,* but not *C_element_extended*.

### Semantic density analyses

**Figure 11:**
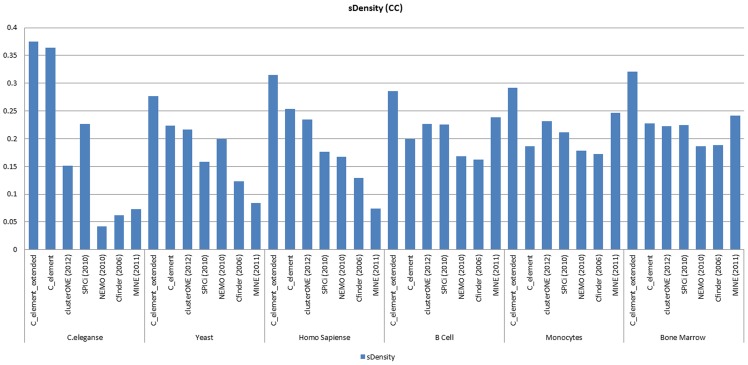
The results of semantic density measure analyses on the results of different algorithms on the six network datasets when *GO CC* derived functional modules were considered.

**Figure 12:**
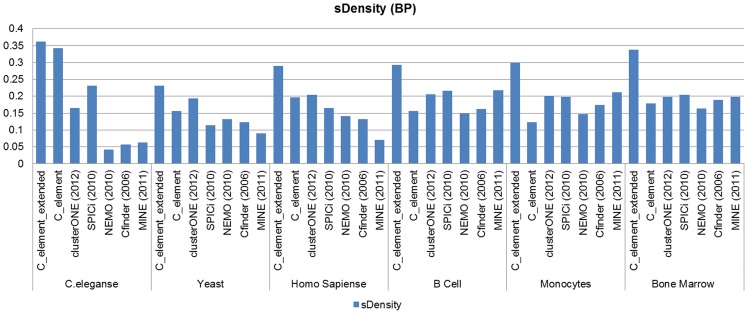
Semantic density measure analyses results on the six different networks and different algorithm considering *GO BP* derived functional modules.

In the last part of our *GO* analyses, we compared different algorithm based on semantic density measure. Figure 11 and Figure 12 show the resulting diagrams based on this measure.

This analysis confirmed our previous two analyses. In *C.eleganse* PPI network *C_element*'s and its extended version's results were appreciable, and on two other networks (*Yeast* and *Human*) *C_element* found *GO CC* derived functional modules better than *clusterONE* and by considering *GO BP* generated functional modules *clusterONE* outperformed *C_element*. In all cases *C_element_extended* outperformed other algorithms. Based on our semantic density analyses, *C_element_extended* also surpassed other algorithms in finding both *GO BP* and *GO CC* derived functional modules on B cells, *Bone marrow CD34* and *Monocytes* tissue specific PPI networks.

### Analyze the effect of using tissue specific networks

The bars illustrated in Figure 7 through Figure 12 also can be used to compare the ability of different algorithms on the tissue specific network datasets to find GO CC derived functional modules and GO BP derived functional modules respectively. Our main goal to use the tissue specific networks was to improve clustering results on *Homo sapiens* PPI network. As it can be inferred from the corresponding bars in 7 through Figure 12, almost all algorithms had a low quality clustering results on *Homo sapiens* PPI network. Using the tissue specific networks, generally all the results of the different algorithms got improved. For example the improvement for *MINE* final results was significant. As it can be inferred from corresponding diagrams in Figure 7 through Figure 12, *MINE* had low quality results on *Homo sapiens*, but the algorithm's results on the other three tissue specific networks were much better. The improvements on the results of *NeMo* and *SPICi* clustering algorithms are also considerable. We can conclude tissue specific networks of human are more accurate and more reliable than the *Homo sapiens* PPI network. The proposed algorithm seemed to have a stable behavior on all six networks. The algorithm found good *GO CC* and *GO BP* derived functional modules in all cases and did not poorly act on any input network.

## Conclusions

In this paper, we focused on finding the high quality functional modules in PPI networks. To fulfill this goal, we have proposed a new clustering algorithm. The new algorithm consists of two main parts. In order to evaluate these two parts, we set up our experiments on the clustering results of each part separately. The *GO* analysis results indicated that the quality of the obtained clusters at the end of the first part of the algorithm (*C_element*) was always much better than *SPICi* and also there was a close competition between the results of the first part of the algorithm and those of *clusterONE*. Based on these analyses we concluded that the obtained seeds were much better than choosing the hubs of the network as the seeds. The first part of the algorithm performed better than all the other algorithms when finding *GO CC* derived functional modules were considered. The analyses on the final clusters at the end of the second part of the new algorithm (*C_element_extended*) showed that the quality of the obtained clusters was significantly better than the quality of the results of the other algorithms on the input datasets. Our analyses also indicated that, generally all the algorithms could find better clusters using tissue specific networks. For example, some algorithms such as *MINE*, *NeMo* and *SPICi* that poorly acted on *Yeast, C.elegans* and *Homo sapiens* PPI networks found considerably better clusters on the tissue specific networks. Generally the new algorithms had a stable behavior on all the input network datasets and did not poorly act on any of them. Therefore, we can conclude that this better performance is not only the result of using tissue specific networks but also the new algorithm plays a part in this performance.

## Supporting Information

File S1 **Contains the sample network from Csermely**'**s original paper and the results of each measure on this network as separate TXT files.**
(RAR)

File S2 **Contains “karate club” network and the results of each measure on this network as separate TXT files.**
(RAR)
